# Diseases of the Circulation

**Published:** 1906-03-24

**Authors:** 


### DISEASES OF THE CIRCULATION.

(*Continued from page 409.*)

**Cardiac Dilatation** is not due, says J. Mackenzie,[7] to the internal pressure forcing the chambers outwards. Even hearts with very thin and degenerate walls may work under high pressure without dilating, and rupture may even take place without any such change. He thinks that the cause is to be found in a depression or lessening of the tonicity of certain portions of the heart muscle. A similar failure of tonic contraction in other bundles of fibres will result in functional murmurs and regurgitation. Thus the loss of tone may affect fibres round the orifices, or attack the walls of the chambers only. Either condition may occur separately, for the bundles of fibres have different functions and different susceptibility to poisons.

**Heart Failure in Diphtheria.**—C. Bolton[8] remarks that in the acute stage this depends on changes in the myocardium and the nucleus of the vagus, but in later stages the voluntary muscles and peripheral nerves, including the vagus, are alone attacked. Therefore in the later stage failure only occurs if some strain or over-exertion happens to the previously weakened heart. The chief remedy is antitoxin, given early enough before the poison has entered into combination with the tissues. Indeed, fatty degeneration of the heart may take place in three days. Secondly, the dose should be large enough, 5,000 units as a minimum, but in severe cases the writer advises 12,000 to 24,000 units at once, and another dose next day if the membrane remains unchanged. The later the time of treatment the larger is the dose required. Antitoxin should be given immediately, without waiting for cultures in any case. Absolute rest is essential, both in the acute and later stages. Death usually happens from heart failure during the second week. There may be slow or rapid pulse with irregularity, a feeble first sound, and accentuated second. Any vomiting or exertion may cause sudden death. He would therefore keep every patient, without exception, in bed for three weeks from the outset, and then only allow getting up if no irregularity or signs of dilatation appear. If these signs do appear they may last for weeks, and the patient should be kept in bed till they do. In extreme cases, after the third month the patient may get up. Late paralyses will not injure the heart unless exertion, vomiting, or respiratory failure increase the strain upon it. As to drugs, digitalis, at least when the pulse is slow, is counter-indicated; so is strychnine, but ether, camphor and alcohol are of some use. Heat or counter-irritation applied to the chest are also valuable. It is claimed that belladonna relieves the over-action of the vagus and aids the heart, but further investigation as to its efficacy is required. T. Nicol [9] reports a case of acute dilatation of the left ventricle six weeks after diphtheria and three weeks after scarlatina in the same patient, a boy aged five. The apex extended three fingers beyond the nipple, but after prolonged treatment the heart's area became normal. The cause of the heart failure was probably an attack of acute nephritis, which raised the vascular tension. The case affords a good instance of the effects of strain on a heart weakened by diphtheria, as described above by Bolton.

**Dropsy.**—F. A. Bainbridge [10] gives an account of recent investigations upon lymph formation and the production of dropsy. Lymph formation is at present held to be due to physical processes, such as osmosis and intercapillary pressure rather than to secretory activity. However, alterations in the capillary endothelium have some share in it. Tissue activity, too, invariably increases lymph production. Dropsy would appear to depend on a primary hydræmic plethora, followed by filtration, but with this we must postulate increased permeability of the capillaries owing to deficient oxidation and impaired nutrition of the capillary walls. Indeed, in heart failure the arteries soon contain less blood than usual, but since the pressure is maintained it is clear that the capacity of the arteries is reduced to the smaller output from the heart. The veins, too, appear to become adapted to the increased blood in them, as shown by some experiments by Bolton, so that there is no great alteration of venous pressure, but probably a small rise both of venous and capillary pressure leading to some œdema. The deficient oxidation leads also to tissue changes and increased diffusion and formation of lymph. There would, then, appear to be three factors at work—increased permeability of vessels, rise of capillary pressure, and increased lymph formation, and of these the first is the most important. J. Mitchell Bruce [11] discusses the value of digitalis in cardiac dropsy, and points out that the diuresis produced by ordinary preparations does not appear for three or four days, and that the drug must be given in full doses. The small, irregular, rapid pulse which may occur during early stages of its administration is not a counter-indication, but points to its continuation, and probably to larger doses. Such a pulse is not a sign of an overdose. When digitalis is causing diuresis, it should not be suddenly reduced or stopped, or we may get a return of the dropsy. In aortic incompetence with dropsy he does not hesitate to give digitalis, though when the pulse becomes very slow it must be reduced, just as in other cardiac affections. Alexander Morison has advocated giving in dropsy up to 30 minims of the tincture every four hours, but he does not seem to have produced diuresis much quicker than Bruce does with 15 minims every four hours. Strophanthin, indeed, acts much more quickly. Eichhorst [12] agrees generally with Bruce as to the value of digitalis in mitral and aortic disease, and also for weakness of the muscle due to affections of the pericardium, coronary arteries and aorta, or to chronic respiratory and kidney diseases. He advises the use of the powdered leaf .1 gramme (1½ grains) three times a day in preference to other preparations. In certain cases he employs small doses for long periods. De Renzi [13] holds that if compensation exists digitalis should not be given, but where compensation has failed it is better than any other drug. Chloëtta's digitalen is, he thinks, the most reliable preparation. A. J. Duprey [14] records a case of remarkable diuresis obtained by the use of Apocynum Cannabinum, but notices the risk of producing nephritis, which excessive doses may cause. In advanced cases of cardiac dropsy R. Crawfurd [15] would prefer to commence with venesection, or, if that cannot be done, with scarification on the dorsum of the feet in order to drain away the fluid freely and quickly. Afterwards digitalis, with or without caffeine, and a dry diet are desirable. Massage enables the skeletal muscles to empty the distended veins, and graduated exercises may be added. If compensation does not take place afterwards the Nauheim baths are likely to be beneficial.

[7] Brit. Med. Jour., Dec. 30. [8] Lancet, Feb. 3 [9] Lancet, Oct. 7. [10] Prac., Nov. [11] Brit. Med. Jour., Jan. 6. [12] Brit. Med. Jour. Epit., Oct. 7. [13] Brit. Med. Jour. Epit., Oct. 14. [14] Lancet, Sept. 30. [15] Prac., Nov.

*(To be continued.)*

